# Positron Annihilation Studies on Chemically Synthesized FeCo Alloy

**DOI:** 10.1038/s41598-018-27949-2

**Published:** 2018-06-27

**Authors:** P. Rajesh, S. Sellaiyan, A. Uedono, T. Arun, R. Justin Joseyphus

**Affiliations:** 1 Magnetic Materials Laboratory, Department of Physics, National Institute of Technology, Tiruchirappalli, 620015 India; 2 Division of Applied Physics, University of Tsukuba, Tsukuba, Ibaraki 305-8573 Japan; 3 Advanced Materials Laboratory, Department of Mechanical Engineering, University of Chile, Santiago, Chile

## Abstract

Equiatomic flower-like FeCo magnetic nanoparticles are synthesized through a modified one-pot polyol technique. The as-prepared samples are annealed at 700 and 800 °C under reducing atmosphere. The saturation magnetization and coercivity of the flower-like FeCo are found to be 198 (1) emu/g and 243 (10) Oe respectively. The magnetic properties of FeCo approach the bulk behavior with annealing. Positron lifetime studies on the chemically synthesized equiatomic FeCo magnetic nanoparticles with flower-like morphology are reported and compared with Fe, Co and FeCo annealed at various temperatures. The FeCo is characterized by different lifetime components corresponding to positron annihilation events in vacancies and various open volume defects due to their unique morphology. The studies suggest defects arising out of cluster vacancies and interpetal gap that reduce on annealing. The average pore size obtained from positron annihilation studies closely matches with the interpetal distance obtained from the electron microscopic analysis for the flower-like FeCo.

## Introduction

FeCo alloys exhibit high saturation magnetization, high Curie temperature and low coercivity that are dependent on the composition as well as order-disorder. FeCo alloy shows ordered B2 (CsCl) type crystal structure (α′) with Fe and Co atoms in interpenetrating simple cubic sublattices^1^. The equiatomic alloy undergoes an order-disorder transformation at 730 °C (T_od_). In the disordered phase (α), the lattice sites are occupied by either Fe or Co atoms randomly, which is obtained on quenching^2^. The α′ phase is brittle due to planar slip and the constraint in the promotion of cross slip. The alloy exhibits antisite and triple point defects in addition to dislocations^3^. Neumayer and Fähnle^4^ investigated the effective formation energies of atomic defects in B2 FeCo by ab initio statistical mechanics and concluded that antisite defect has smaller effective formation energy than vacancies. In stoichiometric B2 FeCo, antisite and triple point defects are minimum^4,5^.

In contrast to the bulk, chemically synthesized equiatomic FeCo magnetic nanoparticles exhibit α phase in the as-prepared state itself^6^. Recently, flower-like FeCo magnetic nanoparticles synthesized through chemical method was also found to exhibit disordered nature^7^. Since chemical method is a low temperature process to obtain the α phase in contrast to the bulk, the structural defects are expected to be different. Positron annihilation lifetime spectroscopy (PAS) is a powerful tool to study atomic defects, in particular, the vacancy type defects and open volume defects of different sizes^8,9^. Due to the high electron density of states, metals and alloys show shorter lifetime component. PAS on single crystal Fe has shown a bulk positron lifetime of 106 ps whereas the lifetime increases to 175 ps for monovacancies^10^. Calculations by Pushka *et al*.^9^ and Ohkubo *et al*.^11^ have shown that the monovacancy lifetime for metals is in the range of 170–180 ps that reaches a saturation value of 400–450 ps when the number of vacancies exceeds nearly 50.

Indeed, the lifetime can also arise from defects other than vacancies. Nanocrystalline Fe was examined by Schaefer *et al*.^12^ and reported the positron trapping in crystalline interfaces and microvoids at the intersection of crystalline interfaces, which could result in lifetimes of 180 ps and 360 ps respectively. Moreover, ortho-positronium (o-Ps) formation on the surface of large voids could result in lifetimes in the 1–140 ns range depending on the void size^13^. The PAS studies on FeCo are scarcely available although other Fe based alloys are reported in the literature^14–19^. These alloys showed the presence of isolated and associated vacancies. PAS on FeCo alloy was studied by Chojcan^20^ and reported that the bulk positron lifetime is nearly 110 ps, which is close to that of Fe.

In dielectric materials, pore size was found to be an important parameter which lowers the dielectric constant. Gidley *et al*.^21^ reported a calculation method in mesoporous thin films to obtain pore sizes in the range of 0.1–600 nm. In bulk metals and alloys, the presence of such large pores is rarely encountered. However, our synthesis method has produced flower-like FeCo with interpetal gaps in the range of 1–10 nm which is sensitive to positron trapping. Depending on the morphology, which could introduce the difference in demagnetization factor, the coercivity of the particles reaches up to 500 Oe^7,22^ due to shape anisotropy whereas the bulk values are less than 10 Oe^1^. The interpetal gaps could also enhance the magnetostatic energy and the formation of multiple domains. The formation of such non-equilibrium structures are facilitated by the defects in the alloy, and therefore PAS can be used as a tool to determine the type of defects present in the alloy. It is also possible to obtain the average interpetal gap in such flower-like morphology using PAS whereas microscopic examination is difficult due to the heterogeneously distributed interpetal gaps. In this paper, we elucidate the positron lifetimes in chemically synthesized equiatomic FeCo and its dependence on the morphology.

## Results and Discussion

### XRD analysis

**Figure 1:**
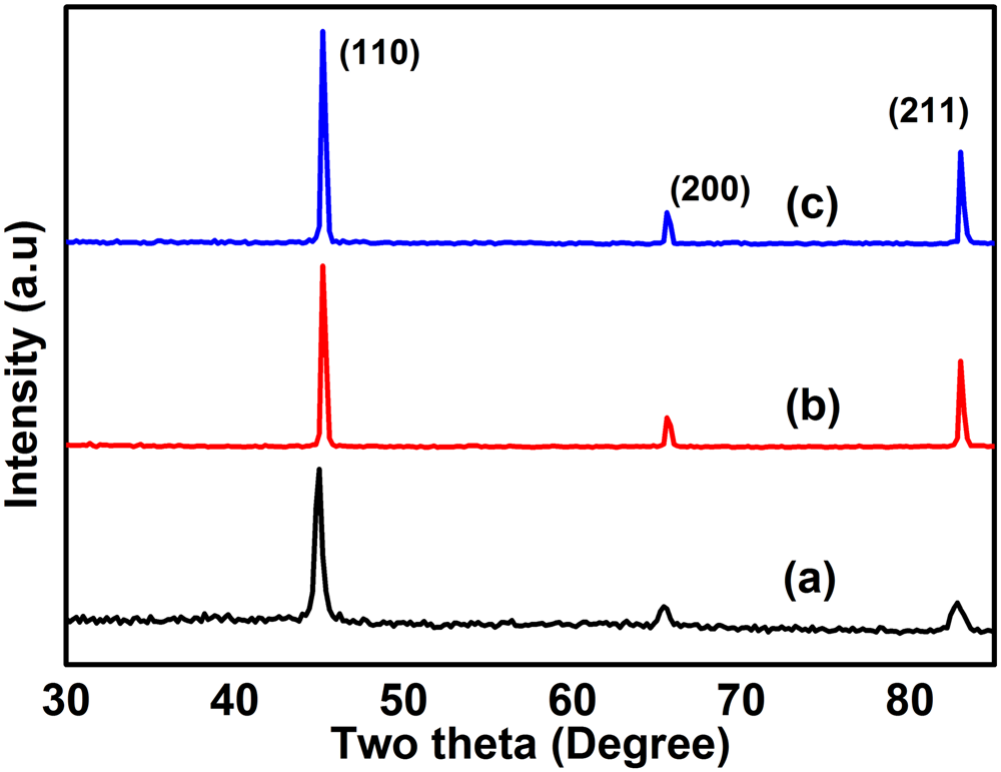
XRD profiles of FeCo (**a**) as-prepared (**b**) 700 and (**c**) 800 °C annealed samples.

Figure 1 shows the X-ray diffraction (XRD) profile of FeCo (a) as-prepared, (b) 700 and (c) 800 °C annealed samples. From the XRD patterns, it was observed that the as-prepared and the annealed samples exhibited bcc FeCo without any impurity phases. The profile consists of three strong diffraction peaks which correspond to (110), (200) and (211) crystal planes. The lattice constant value was found to be 2.852 (5) Å which is in close agreement with the value of 2.848 Å corresponding to bulk FeCo^1^.

### Microscopic analysis

**Figure 2:**
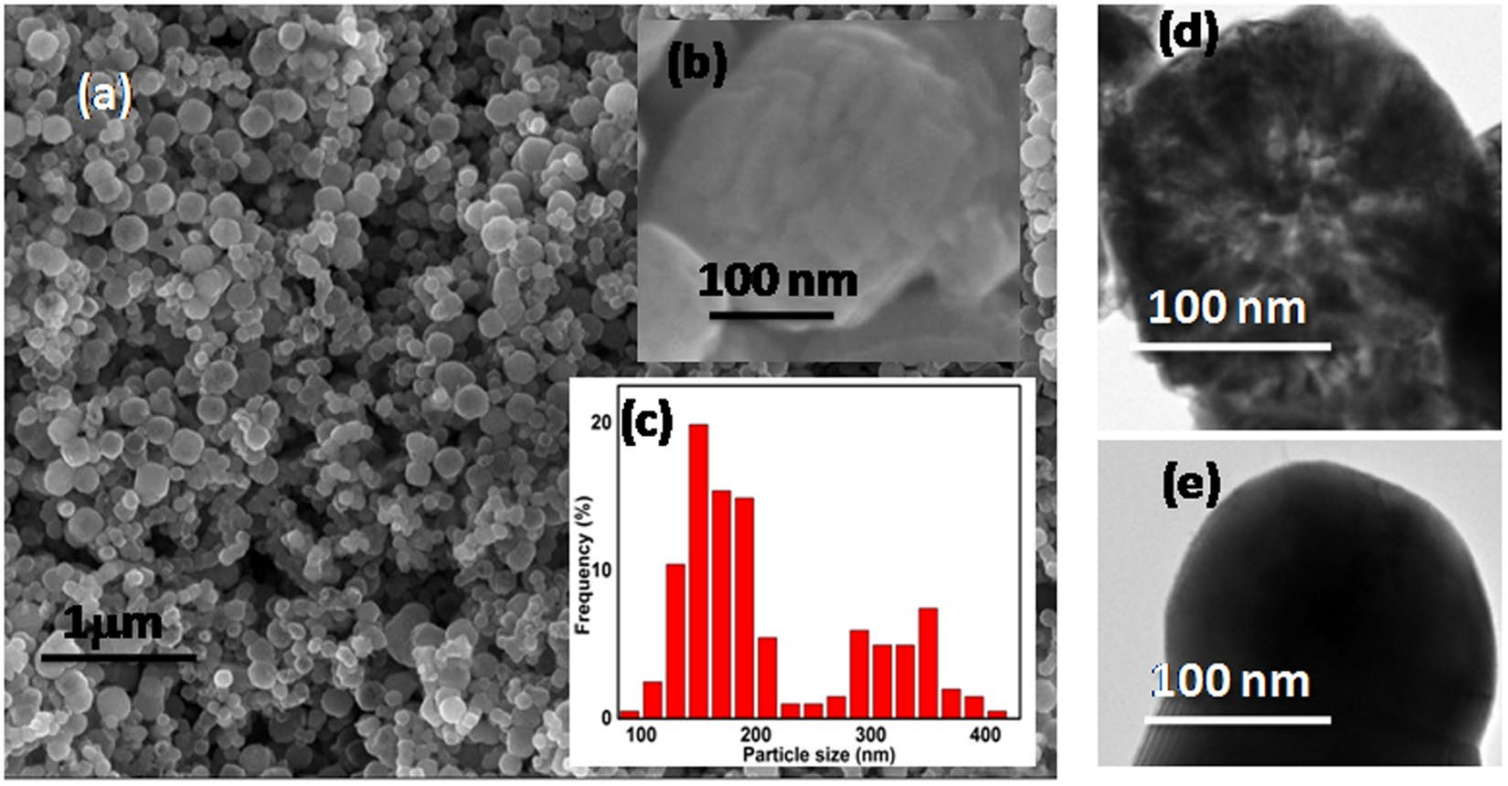
FESEM image of FeCo (**a**) as-prepared (**b**) magnified image and the (**c**) particle size distribution; TEM images of FeCo (**d**) as-prepared and (**e**) annealed at 700 °C.

Figure 2 shows the scanning electron microscopy (SEM) (a) image of the as-prepared FeCo particles, (b) the higher magnification image and (c) the histogram plot showing the size distribution of the particles. The particle size was found to be in the range of 100–400 nm. It is apparent from the SEM (Fig. 2(b) insert) that the particles have flower-like shape. The petals are laminated one above the other to form a spherical ball-like structure. Irrespective of the particle size, the flower-like structure was present in all the samples. The interpetal distance as found from SEM is in the range of 9–13 nm. Figure 2(d,e) shows the TEM images of as-prepared FeCo and the same annealed at 700 °C respectively. The petals could be clearly seen from the TEM image (Fig. 2(d)) for as-prepared FeCo. The interpetal distance was found to be 8–12 nm which is in agreement with the SEM results. However, on annealing at 700 °C, the morphology is lost and the flowers agglomerate to obtain larger particle as shown in Fig. 2(e).

**Figure 3:**
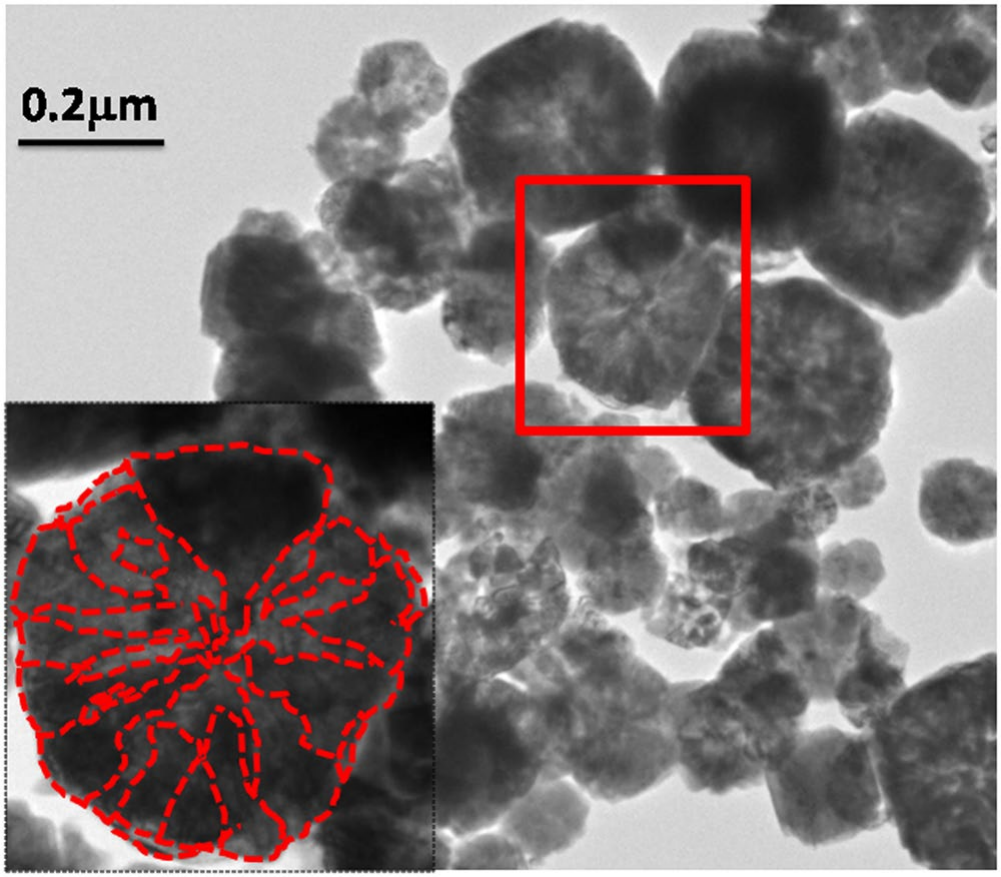
TEM image of as-prepared FeCo. Inset shows the outline of flower-like petals.

Figure 3 shows the TEM micrograph of the as-prepared FeCo sample with most of the particles exhibiting a flower-like arrangement. The inset shows one such FeCo flower with the petals marked as contours. The interpetal gap could be visible as lighter shades in the grey scale image. It is to be noted here that the experimental conditions have resulted in more number of petals and gaps in the form of pores compared to our previous study^7,22^. Although the flower-like morphology is retained, the number of petals and the interpetal gap is found to vary due to the nucleation and growth rates, depending on the local experimental conditions in the synthesis medium (polyol). Fe rich FeCo is found to exhibit well separated flower-like petals^22^ whereas more petal-like morphology is encountered in the present samples. The arrangement of petals in equiatomic FeCo is determined by the growth of initial truncated polyhedral nuclei.

### Magnetic properties

#### Thermomagnetic analysis

**Figure 4:**
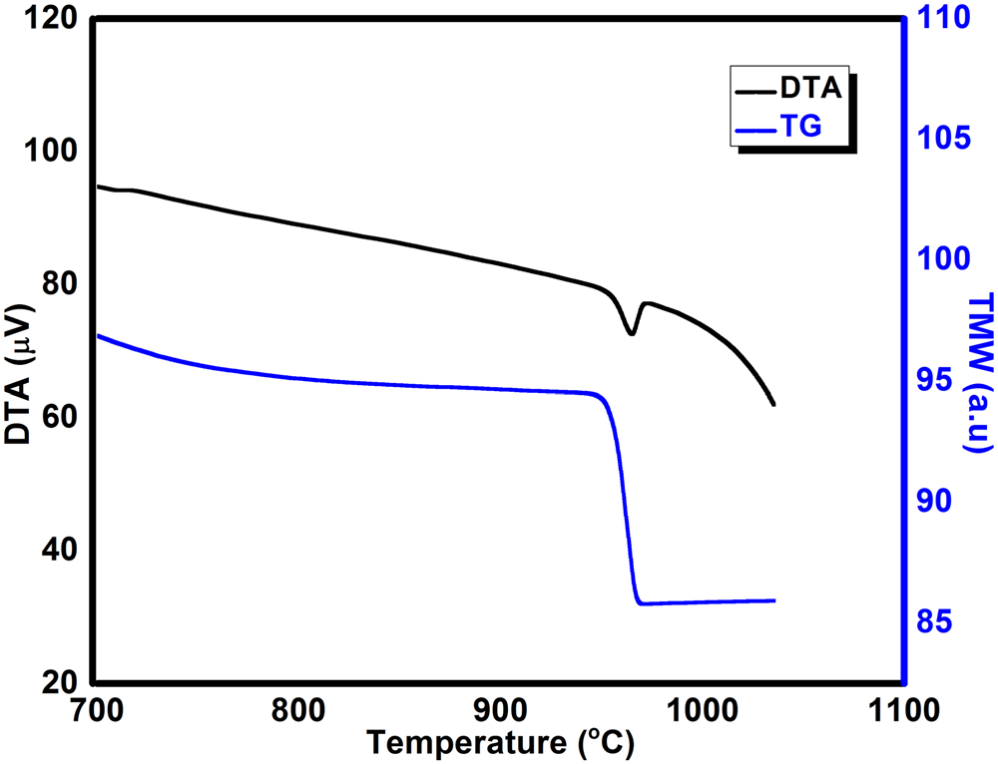
Thermomagnetic weight loss and DTA profile of as-prepared FeCo.

Figure 4. shows the thermomagnetic and DTA profiles of as-prepared FeCo. The T_c_ for the as-prepared sample was measured to be 965 °C from the thermomagnetic weight loss which is close to that of bulk FeCo (970 °C). The phase transition temperature T_α→γ_ at which the α phase transforms to γ phase was measured to be the same as that of T_c_. The coincidence of the T_c_ and T_α→γ_ indicates the equiatomic composition of FeCo^23^.

#### Hysteresis loop measurement

**Figure 5:**
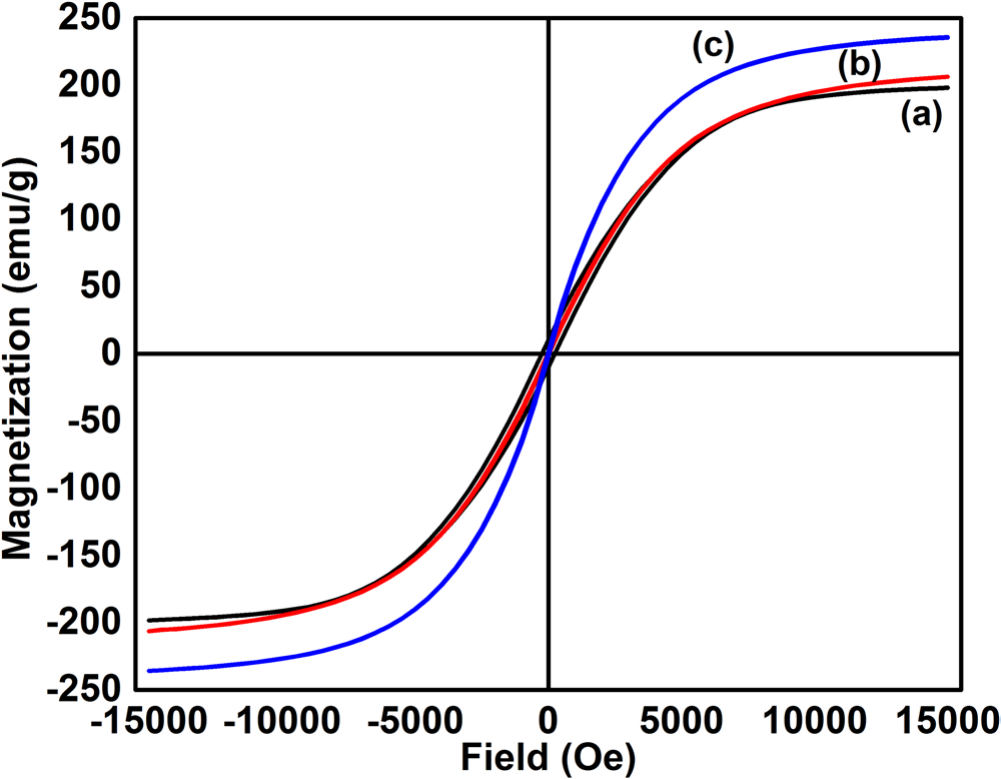
Room temperature hysteresis loops for the as-prepared (**a**) and annealed samples (**b**,**c**).

The room temperature hysteresis loop measurement for the as-prepared (a) and annealed samples (b-c) is shown in Fig. 5. The saturation magnetization (M_s_) and coercivity for the as-prepared samples are 198 (1) emu/g and 243 (10) Oe respectively. The M_s_ increases to 236 (1) emu/g with the increase of annealing temperature to 800 °C which suggests the removal of oxide layer while annealing under reducing atmosphere. The coercivity reduces to 26 (1) Oe indicating the transformation towards the bulk value. Here it is to be emphasized that high coercivity (500 Oe) could be obtained in FeCo with well-separated petals^22^ whereas it reduces to 243 Oe in our present samples. Thus, the presence of interpetal gap, which is also dependent on the number of petals, plays an important role in obtaining shape dependent magnetic properties.

### Defect studies

In PAS, the change of electron density in the vicinity of defects will result in the lifetime variation of the annihilating positrons that forms the basis for defect identification. The lifetime component can then be used to find out the type of vacancies and other open volume defects which are present even in bulk materials. Since Fe and Co are the base elements of FeCo alloy, we have studied the PAS of powders and rods of Fe and Co, chemically synthesized FeCo and samples annealed at 700 and 800 °C. The Fe and Co powders are named as Fe-P, Co-P respectively and the rods are named as Fe-R, Co-R. The positron lifetime components (*τ*) with corresponding intensities (I) for different materials under study are given in Table 1. The PAS study resulted in three lifetime components for all the samples except for Fe-R, Co-R and as-prepared FeCo (FC-AP). For the FC-AP, reasonable fitting could be achieved only with four lifetime components. The mean lifetime (*τ*_m_) for each sample was calculated using the relation (*τ*_1_I_1_ + *τ*_2_I_2_)/(I_1_ + I_2_).

**Table 1:** Positron lifetimes with corresponding intensities for Fe, Co and FeCo.

Sample details	*τ*_1_(ps)	I_1_%	*τ*_2_ (ps)	I_2_%	*τ*_3_ (ps)	I_3_%	*τ*_m_ (ps)
					*τ*_4_ (ns)	I_4_%	
Fe powder	158 (4)	63 (3)	323 (18)	33 (2)	622 (55)	4 (1)	215
Co power	145 (6)	61 (6)	274 (26)	31 (4)	514 (27)	8 (2)	188
Fe rod	140 (1)	86 (1)	268 (6)	14 (1)	0	0	158
Co rod	156 (1)	95 (1)	283 (19)	5 (1)	0	0	162
FeCo as prepared	180 (10)	9.5 (1)	345 (1.1)	87.5 (1)	1852 (172)	0.5 (0.01)	329
					54 (0.4)	2.5 (0.1)	
FeCo annealed at 700 °C	133 (7)	38 (2.8)	284 (6.7)	59 (2)	698 (31)	3 (0.5)	225
FeCo annealed at 800 °C	133 (2.6)	59.5 (1.5)	292 (4.7)	39.6 (1)	959 (61)	0.9 (0.1)	197

From Table 1 it could be noticed that the *τ*_1_ for Fe and Co are in the range of 140–158 ps for both the powder and rod samples. These values are larger than the bulk positron lifetimes of Fe (106 ps) and Co (118 ps)^10,24^. Since the monovacancy lifetime for Fe is above 175 ps from calculation and experiments^9^, the lower *τ*_1_ suggests positron trapping in dislocation defects other than vacancies. The calculated lifetime of vacancy on edge dislocation line in Fe is found to be 140 ps and that of divacancy on edge dislocation is 150 ps^25,26^. Schaefer *et al*.^12^ showed that interfacial defects exhibit lifetime around 180 ps in nanocrystalline Fe. Therefore the observed *τ*_1_ with lifetime larger than the bulk but below the monovacancy and interfacial defects in our studies could be assigned to originate from the vacancies associated to edge dislocations. The *τ*_1_ for Co-P and Co-R was found to be 145 and 156 ps respectively from our studies, which is larger than the bulk lifetime of 118 ps^24^. Considering the lifetime of 175 ps for monovacancy in Fe, cobalt is expected to show a higher lifetime than its bulk^27^. However, the *τ*_1_ value between the bulk and monovacancy lifetime suggest that associated vacancy is the contributing factor, similar to Fe.

The *τ*_2_ for Fe-P and Fe-R are 323 and 268 ps with intensities (I_2_) 33 and 14% respectively. From the theoretical and experimental lifetime values for Fe^9^, the *τ*_2_ for Fe-P could be easily assigned to 9-10 vacancies. The *τ*_2_ value of 268 ps for Fe-R could emanate from 4-5 vacancies. From the comparison of *τ*_2_ for Co and other reported metals^9^, it could be consigned to cluster vacancy. The higher I_2_ for both Fe-P and Co-P compared to Fe-R and Co-R respectively indicate the presence of more cluster vacancies in the micron-sized powders. The cobalt powder is fitted with three lifetime components whereas the Co rod is characterized by two lifetime components. The third lifetime (*τ*_3_) for Fe and Co powders exhibits a value higher than cluster vacancy lifetimes^9,11^ with least intensity and therefore it may be due to positron trapped in pores, arising out of the packing of the powder samples. From the studies on Fe and Co, it is clear that edge dislocation defects and cluster vacancies are present in both powders and rods.

The FC-AP sample showed four lifetimes whereas FC-700 and FC-800 showed three lifetimes. It is to be recollected here that FC-AP sample exhibits flower-like morphology whereas the morphology is lost on annealing. The lifetime *τ*_1_ showed a higher value of 180 ps with a lower intensity of 9.5% in contrast to the powders and rods of Fe and Co. This lifetime is assigned to the isolated monovacancy defect contributions. Also, a careful examination of the *τ*_1_ for FC-700 and FC-800 reveals that the intensity increases with annealing temperature suggesting their transformation towards the bulk behaviour.

**Figure 6:**
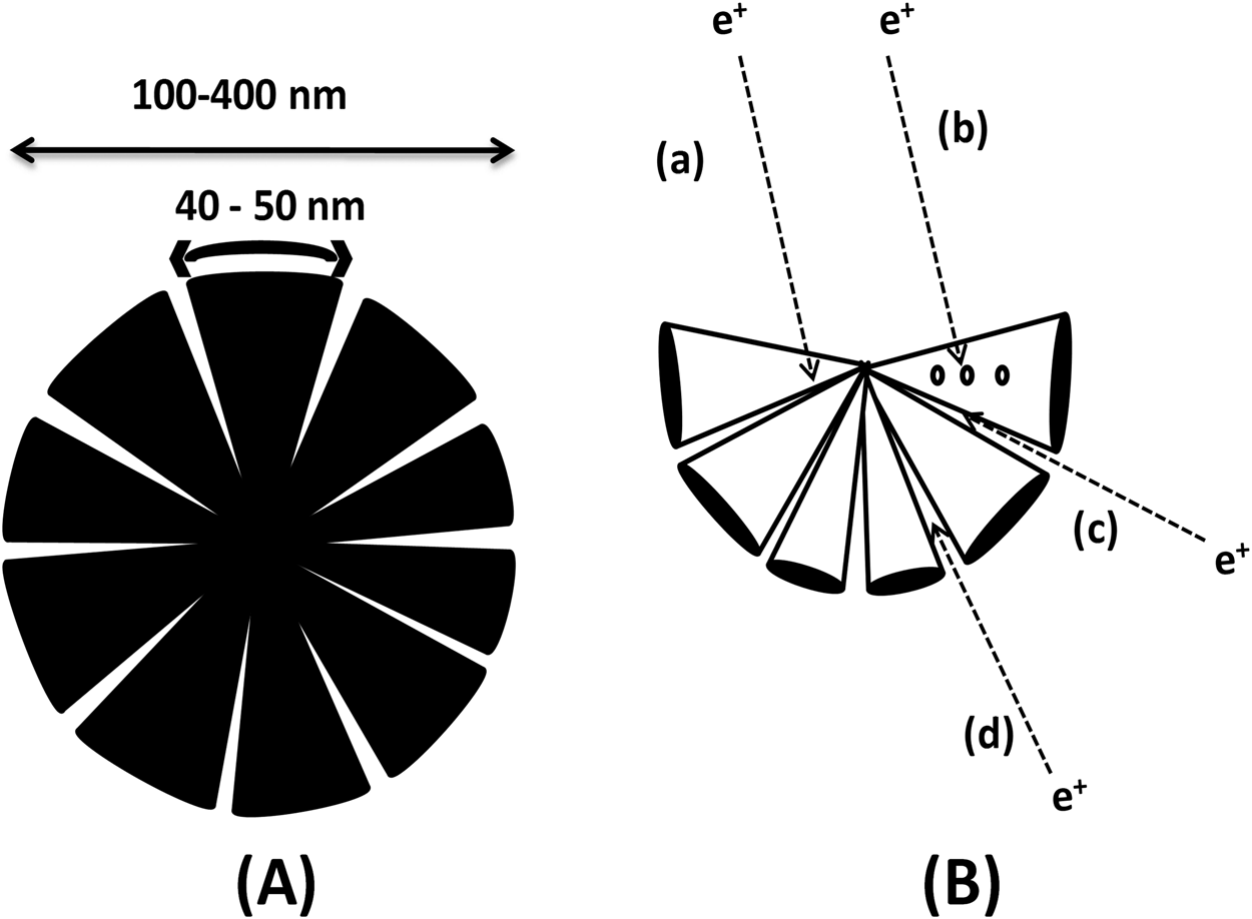
(**A**) Schematic diagram of the flower-like morphology (**B**) positron trapping at the open volume defects.

The positron annihilation at various defect regions in the flower-like particles is illustrated in Fig. 6. Figure 6(A) shows the schematic of the FeCo with flower-like morphology and 6 (B) shows the possible defects where annihilation events occur. Figure 6(A) represents the dimensions of the particle including the interpetal gap. The morphology can be modeled close to that of a wedge-shaped interpetal gap inferred from the SEM and TEM images. The interpetal gap opens with sizes around 8–13 nm, as obtained from microscopy analysis, and narrows down towards the centre. Positron trapping occurs at various locations in the wedge-shaped open volume defect. From Fig. 6(B), various trapping sites could be identified (a) associated and monovacancy (b) cluster vacancies in the FeCo alloy (c) narrow wedged interpetal gap and (d) wide interpetal gap.

The *τ*_2_ and I_2_ of FC-AP are 345 ps and 87.5% respectively. From our previous discussions, the equivalent number of vacancies corresponding to 345 ps lies in the range of 10–15. The mean lifetimes for all the samples except FC-AP lie in the range of 158–225 ps. The large *τ*_m_ (329 ps) for FC-AP suggests that more open volume defects are present in the chemically synthesized FeCo alloy. This could be corroborated by the fact that *τ*_m_ decreases with annealing temperature resulting in 225 and 197 ps for FC-700 and FC-800 respectively, suggesting reduction of defects. The *τ*_3_ component for FC-700 now becomes equivalent to the *τ*_3_ of Fe-P due to the transformation towards the bulk.

The mean free path and diffusion length of positron in metals are in the range of 10 and 100 nm respectively. Considering a range of open volume defects due to the wedge-shaped interpetal gap, the *τ*_3_ and *τ*_4_ values of 1.852 ns and 54 ns with intensities 0.5 and 2.5% respectively for FC-AP could be assigned to pick-off annihilation of ortho-positronium (o-Ps). The o-Ps lifetime could extend above 0.5 ns^12^ and the much larger *τ*_4_ is exclusively from the morphology since our studies with a closed petal shape showed the disappearance of the fourth lifetime component. Also, the *τ*_4_ vanishes with annealing temperature as observed for FC-700 and FC-800 where the morphology is completely lost.

**Figure 7:**
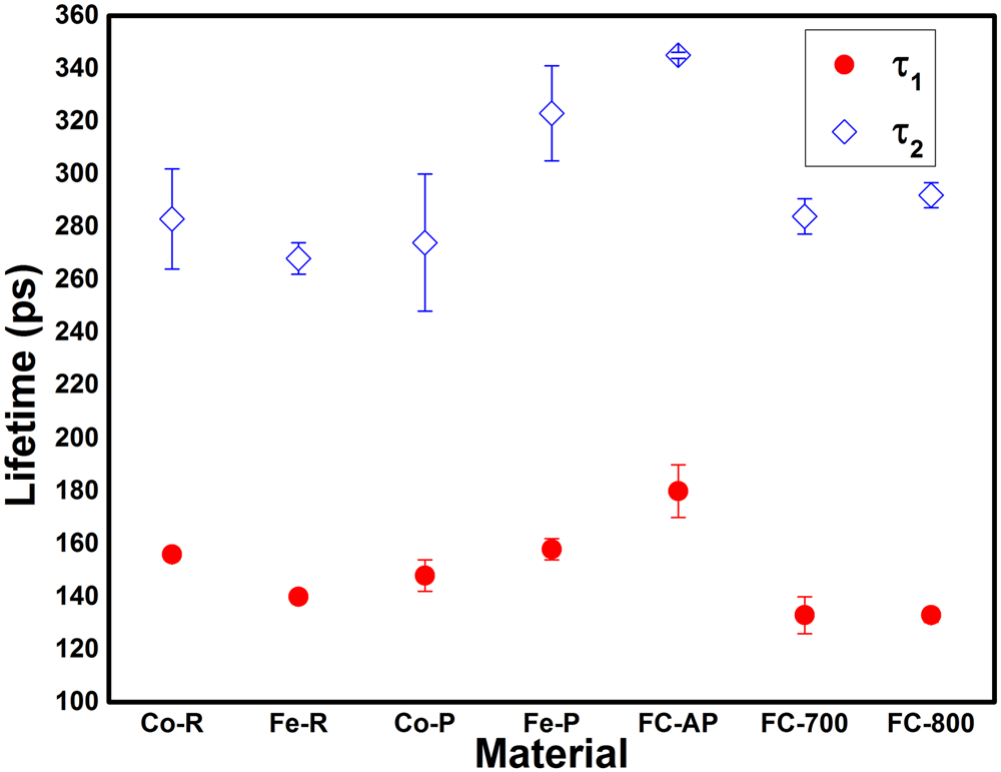
Positron lifetimes for different materials.

**Figure 8:**
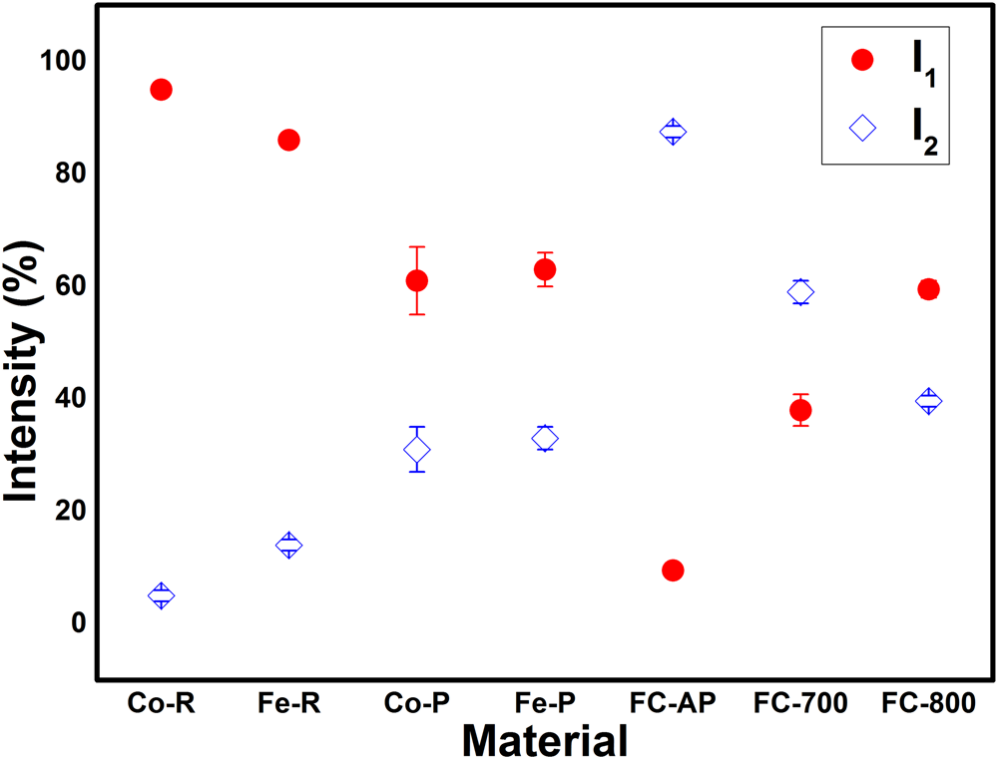
Lifetime Intensities for different materials.

Figures 7 and 8 shows the positron lifetimes (*τ*_1_ and *τ*_2_) and their corresponding intensities (I_1_ and I_2_), respectively for the various materials under our study. The lifetime components, *τ*_1_ and *τ*_2_ for the as prepared FeCo are 180 and 345 ps respectively which are clearly higher than that of all the samples as shown in Fig. 3. Paulin *et al*.^28^ reported *τ*_1_ and *τ*_2_ of 200 and 450 ps respectively for Fe, Co and FeNi powders and concluded that positron trapping occurs in metal surface oxide layer. In the present studies we have not observed such a large lifetime in FC-AP although an oxide layer may be present. The thin layer of oxide in FC-AP may be below 2 nm in size^7^, which is much smaller than the positron diffusion length scale. It is to be noted here that these two lifetimes decrease with annealing temperature as observed for FC-700 and FC-800. The *τ*_1_ for the annealed FeCo is the least among all the samples indicating their transformation towards bulk as discussed below.

The lifetime component *τ*_1_ of FC-700 and FC-800 is 133 ps which is greater than the bulk positron lifetime but lesser than the monovacancy lifetime. This result is in accordance with the study by Seegers *et al*.^29^ suggesting that the lattice defects smaller than monovacancy are also effective positron traps. Moreover, defects associated with dislocations such as jogs can also act as positron trapping sites^30^. The positrons are sensitive to the length and structure of jog^31,32^. Nevertheless, the lifetime for jog on edge dislocation was calculated to be 117 ps by Kamimura *et al*.^25^. Therefore, the first life time in annealed FeCo can be attributed to the average lifetimes of the bulk and the associated vacancies. Such associated vacancies have been reported in other Fe based alloys^18,19^. The lifetime component *τ*_1_ for FC-800 is same as that of FC-700 but the former has greater intensity. The intensity increases for *τ*_1_ with annealing temperature whereas it decreases for the other lifetime component (*τ*_2_) as shown in Fig. 7. The concentration of cluster vacancies and microvoids are significantly reduced with annealing temperature and the small clusters are recovered completely resulting in increasing I_1_ without affecting *τ*_1_. Lifetimes similar to clusters can be also contributed by the interpetal interface in FC-AP. Concurrently the closure of the petals with annealing temperature could result in elimination of interfacial defect there by lowering the lifetime *τ*_1_ and increasing the I_1_.

The slight increase in *τ*_2_ for FC-800 can occur due to the averaging of the lifetime components. The large interpetal interfaces are reduced giving lifetime equivalent to clusters with higher annealing temperature. As the petal-shaped morphology is lost with annealing, it is also clear that the large interpetal interface does not exist and therefore the intensity (I_2_) reduces with annealing as inferred from Fig. 8. The annealed samples are in the B2 phase since 700 °C lies in the temperature region of order-disorder transformation. The lifetime from open volume defects is considerably large compared to the antisite defect^33^. Therefore, from the first lifetime, we could not distinguish between the order-disorder dependent lifetimes for the as-prepared and annealed samples. Moreover, studies by Wolff *et al*.^17^ and Diego *et al*.^34^ in FeAl have shown that there is no significant variation in the lifetimes between the A2 and B2 structure compared to vacancies.

Lifetimes in the nanosecond range could give information about the pores from the localized positronium formation. Positronium (Ps) is a quasi-stable neutral bound state of positron and electron which exists in a singlet state (p-Ps) and triplet state (o-Ps) with spins zero and one respectively. In vacuum, o-Ps has a lifetime of 142 ns^35^ that reduces according to the pore size. The o-Ps lifetime can be estimated from the decay constant using the expression $\lambda ={\lambda }_{T}+{\lambda }_{P}$ and ${\lambda }_{P}=\frac{P}{\ell }$ where λ_T_, λ_P_, P and $\ell$ are the o-Ps vacuum decay rate, decay rate in pores, diffusion constant and mean free path between collisions of Ps respectively^21^. The extension of Tao-Eldrup model using a rectangular model (RTE model) can be used to find the Ps decay rate in pores of any size and aspect ratio. The pore size can be calculated from the RTE model given by Gidley *et al*.^21,36^ using the following relation

$$\begin{array}{c}{\lambda }_{{\rm{RTE}}}(D,T)={\lambda }_{{\rm{A}}}-\frac{{\lambda }_{s}-{\lambda }_{T}}{4}{[1-\frac{2\delta }{D}+\frac{\sum _{i=1}^{\infty }\frac{1}{i\pi }\sin (\frac{2i\pi \delta }{D}){e}^{(\frac{-\beta {i}^{2}}{{D}^{2}KT})}}{\sum _{i=1}^{\infty }{e}^{(\frac{-\beta {i}^{2}}{{D}^{2}KT})}}]}^{3}\\ {\lambda }_{A}=\frac{({\lambda }_{S}+3{\lambda }_{T})}{4};\,\beta =\frac{{h}^{2}}{16m}\end{array}$$

where λ_S_, δ, D, h, m, T are the p-Ps decay rate, geometry dependent wall thickness, pore size, Planck constant, mass of the electron and absolute temperature respectively. Using δ = 0.19^37^, the pore size was calculated to be close to 0.9 and 4.2 nm from the third and fourth lifetime components for FC-AP. These values indicate that the positron encounters an average pore size with surrounding electron density due the complexity associated with the interpetal distance. The pore size of 4.2 nm is close to the lower limit in the average interpetal gap of 8–13 nm observed from SEM and TEM. The interpetal gap arising out of the flower-like morphology is responsible for the higher average lifetime of the as-prepared FeCo particles.

## Conclusion

Positron lifetime spectroscopy of chemically synthesized equiatomic FeCo with flower-like morphology has been studied. The interpetal gap arising out of the flower-like morphology act as effective positron trapping centers leading to the pick-off annihilation of positronium. These morphological defects act as pores which give rise to longer lifetimes of 1.8 and 54 ns from two different average pore sizes. The average pore size calculated using the rectangular extension of Tao-Eldrup model from the fourth lifetime is  4.2 nm. The fourth lifetime component disappears on annealing due to the loss of flower-like morphology. The FeCo annealed at temperatures above 700 °C exhibited *τ*_1_ of 133 ps which is shorter than the lifetime of their constituent elements due to vacancies associated with dislocation.

### Experiment

High purity Fe (99 + %) and Co (99.9%) powders and Fe (99.95 + %) and Co (99.98%) rods of φ9.5 × 100 mm and φ5.0 × 30 mm respectively were purchased from The Nilaco Corporation Japan. Equiatomic FeCo magnetic nanoparticles were synthesized based on one pot polyol process^22^ using ethylene glycol as a reducing agent. Iron (II) chloride tetrahydrate (FeCl_2_.4H_2_O) and cobalt acetate tetrahydrate (Co (OAc)_2_.4H_2_O) precursors of equal molarity (total molar ratio 0.1 mol/L) were weighed and physically mixed together. Then, they were introduced into ethylene glycol at 190 °C under constant stirring. NaOH pellets were incorporated into the solution after 5 s resulting in a black precipitate of FeCo which was recovered using magnetic separation. The as-prepared FeCo powders were annealed at 700 and 800 °C in a reducing atmosphere of 97% Ar + 3% H_2_ with a heating rate of 30 °C/min. The samples were furnace cooled to room temperature in a duration of 10 h. The as-prepared and samples annealed at 700 and 800 °C are named as FC-AP (FC stands for FeCo, AP stands for as-prepared), FC-700 and FC-800 respectively.

X-ray diffraction patterns of FeCo were recorded using the Ultima III Rigaku X-ray diffractometer with conventional Cu Kα radiation. Thermomagnetic analysis with an external magnet was carried out using EXSTAR TG/DTA6200 under Ar atmosphere with the flow rate of 500 ml/min. The composition was also confirmed from the EDX (Energy dispersive X-ray) analysis. The room temperature hysteresis loop measurement was recorded using vibrating sample magnetometer (VSM) model 7404, Lakeshore, USA. The morphological properties of the samples were investigated using Zeiss, Neon 40FESEM scanning electron microscope (SEM) and JEOL 2010- 200 kV transmission electron microscope (TEM).

Positron lifetime measurements were carried out using a conventional lifetime spectrometer with digital oscilloscope with a full width at half maximum time resolution of ~190 ps. The number of lifetime components is determined by deconvoluting the spectra, with each spectrum having counts above 10^6^_,_ after performing source and background correction. Two copper plates each of dimension 15 × 15 × 3 mm were grooved at the center by 7 mm diameter. Then the ^22^NaCl source is sandwiched between the copper plates in the grooved region. The powder sample is placed at the groove on both sides and closed with the kapton tap. For the measurement of rod samples, two pieces of bulk samples from Fe rod (9.5 mm diameter and 2 mm thickness) and Co rod (5 mm diameter and 2 mm thickness) were sliced and the source is sandwiched between the samples.
